# Serum β‑hCG as a prognostic marker for colorectal adenocarcinoma in women of reproductive age: A case report and literature review

**DOI:** 10.3892/mi.2024.207

**Published:** 2024-11-18

**Authors:** Changlin Gong, Fnu Vikash, Juliet Silberstein, Sherrie White, Donald Kotler

**Affiliations:** 1 Department of Medicine, Jacobi Medical Center, Albert Einstein College of Medicine, Bronx, NY 10461, USA; 2 Department of Pathology, Jacobi Medical Center, Albert Einstein College of Medicine, Bronx, NY 10461, USA; 3 Division of Gastroenterology, Jacobi Medical Center, Albert Einstein College of Medicine, Bronx, NY 10461, USA

**Keywords:** colorectal adenocarcinoma, free βsubunit of human chorionic gonadotropin, tumor marker

## Abstract

Serological and histological examinations have unveiled the presence of free β subunit of human chorionic gonadotropin (β-hCG) in some cases of colorectal adenocarcinoma. The present study describes an unusual case of a young female patient afflicted with rapidly advancing adenocarcinoma of the sigmoid colon. Despite the absence of pregnancy indications on an ultrasound and curettage, a notable elevation of β-hCG was detected in the serum, alongside positive staining in the tumor tissue. β-hCG-positive colorectal adenocarcinoma is associated with a lower survival rate, tissue invasion and metastases, as well as a good response to chemotherapy, compared with β-hCG-negative counterparts. Moreover, the serum β-hCG level decreases with treatment. Thus, β-hCG may serve as a tumor marker for response monitoring. However, further studies are required to investigate the potential benefit of routine serological and histological β-hCG studies in patients with colorectal adenocarcinoma.

## Introduction

Oncofetal tumor markers associated with colon cancer has been extensively studied and elevated levels of carcinoembryonic antigen (CEA) and carbohydrate antigen (CA) 19-9 (CA 19-9) have been found to be associated with a worse prognosis (1). In clinical practice, CEA and CA 19-9 levels are usually monitored in patients with colon cancer. In addition, other serum tumor markers are also associated with a poor prognosis, including CA 242, CA 72-4 and free β subunit of human chorionic gonadotropin (β-hCG) in both males and females (2,3). However, the cut-off value for β-hCG as a negative prognostic factor for colon cancer in a previous study was very low (2 pmol/l, 1 IU/l=2.93 pmol/l) (4). Furthermore, the detection of β-hCG in biopsy samples has also been shown to be associated with deeper tissue invasion, lymph node and liver metastases, and a lower survival rate (5-7). Previously reported β-hCG-positive colon cancer was mostly identified in elderly patients; however, in females of reproductive age, positive serum levels of β-hCG pose a unique challenge in differential diagnosis for clinicians. The present study describes the case of a young female patient with colon cancer and very high serum β-hCG levels.

## Case report

**Figure 1:**
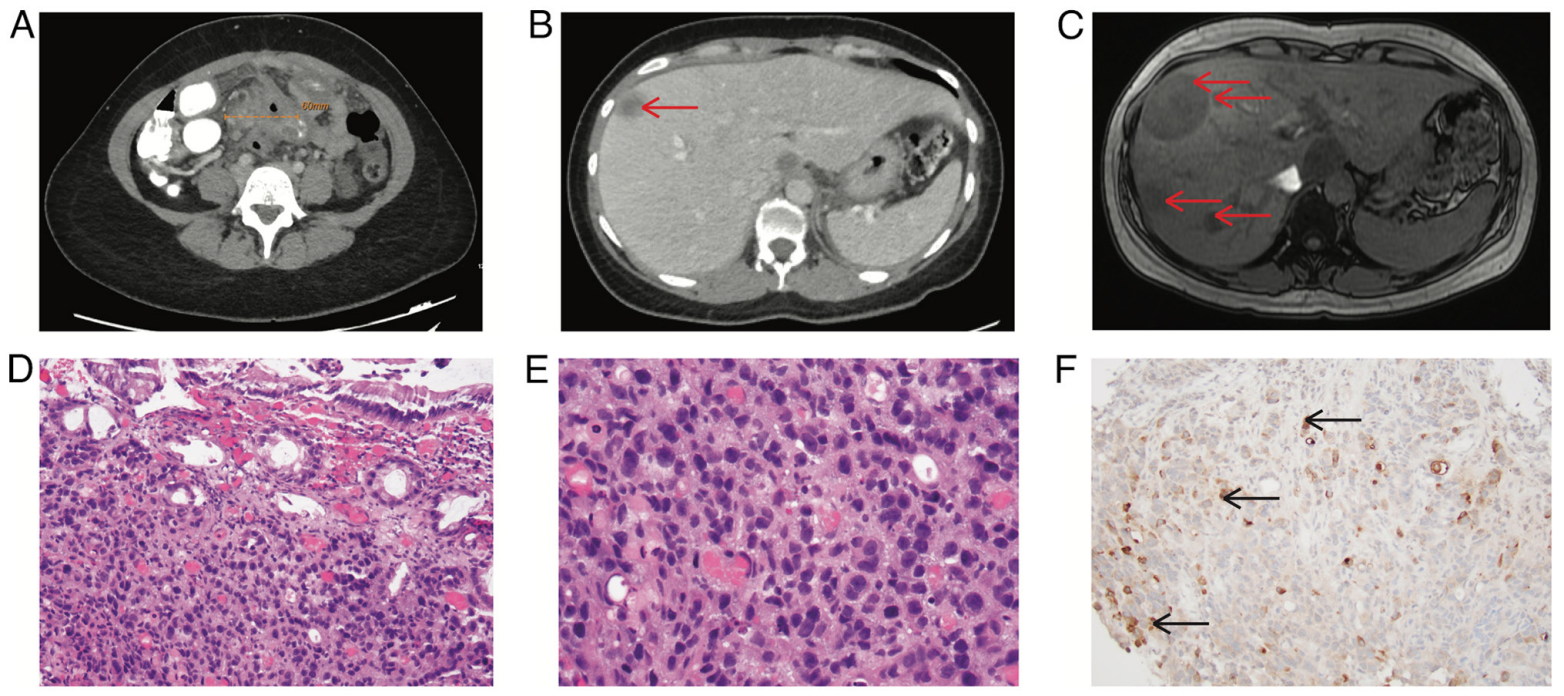
Abdominal imaging studies. (A) Abdominal CT scan upon first presentation, illustrating mid-abdomen collection. (B) Abdominal CT scan upon first presentation, illustrating liver lesions. (C) Abdominal MRI 1 month later, demonstrating worsened liver lesions. (D) Hematoxylin and eosin staining of biopsy sample (magnification, x20). (E) Hematoxylin and eosin stain of biopsy sample (magnification, x40). (F) β-hCG staining of biopsy sample. Red arrows in panels B and C indicate liver lesions; black arrows in panel F indicate some of the β-hCG-positive cells. β-hCG, free β subunit of human chorionic gonadotropin.

A 30-year-old female, G2P1, who presented to the Emergency Department of Jacobi Medical Center in Bronx, New York, USA, 14 months following delivery, with nausea, vomiting and severe abdominal pain for 2 weeks. She had occasional bright red blood per rectum for 4 months and had experience a weight loss of 5 kg in 2 weeks. An abdominal computed Tomography (CT) scan revealed an ill-defined collection in the mid-abdomen, measuring up to 6 cm, with adjacent thickened loops of the small bowel, as well as the sigmoid colon (Fig. 1A) and hypodense lesions in the liver (Fig. 1B). A colonoscopy revealed mucosal congestion and narrowing in the sigmoid colon, which could not be traversed with the colonoscope. However, a biopsy at the narrowing area revealed only colonic mucosa. She continued to have mild abdominal pain and serum β-hCG was detectable at 5.9 IU/l (normal range, 0.02 to 0.8 IU/l) 1 month later. An abdominal MRI instead of a CT scan was performed for concerns of pregnancy. This revealed more severe multiple hepatic lesions (Fig. 1C). A liver biopsy, obtained 1.5 months later, revealed moderately differentiated adenocarcinoma with abundant necrosis. Serum tumor markers were tested at that time, with borderline CEA levels (5.4 ng/ml; upper limit of normal, 5.0 ng/ml) and significantly elevated CA19-9 levels (1,168 U/ml; upper limit of normal, 35 U/ml). Immunohistochemical analysis was performed by GenPath Bioreference Laboratories (Elmwood Park, NJ, USA). This laboratory is certified under the Clinical Laboratory Improvement Amendments of 1988 (CLIA-88), CLIA number 33D0668554. Biopsy samples were paraffin-embedded and the thickness of the sections was 4 µm. The results of immunohistochemical staining under light microscope were consistent with colorectal origin (data not shown). Repeat colonoscopy re-demonstrated severe stenosis in the sigmoid colon with abnormal appearing mucosa. An endoscopic biopsy revealed poorly differentiated adenocarcinoma (Fig. 1D and E), with the immunohistochemistry stains consistent with colonic adenocarcinoma (data not shown).

**Figure 2:**
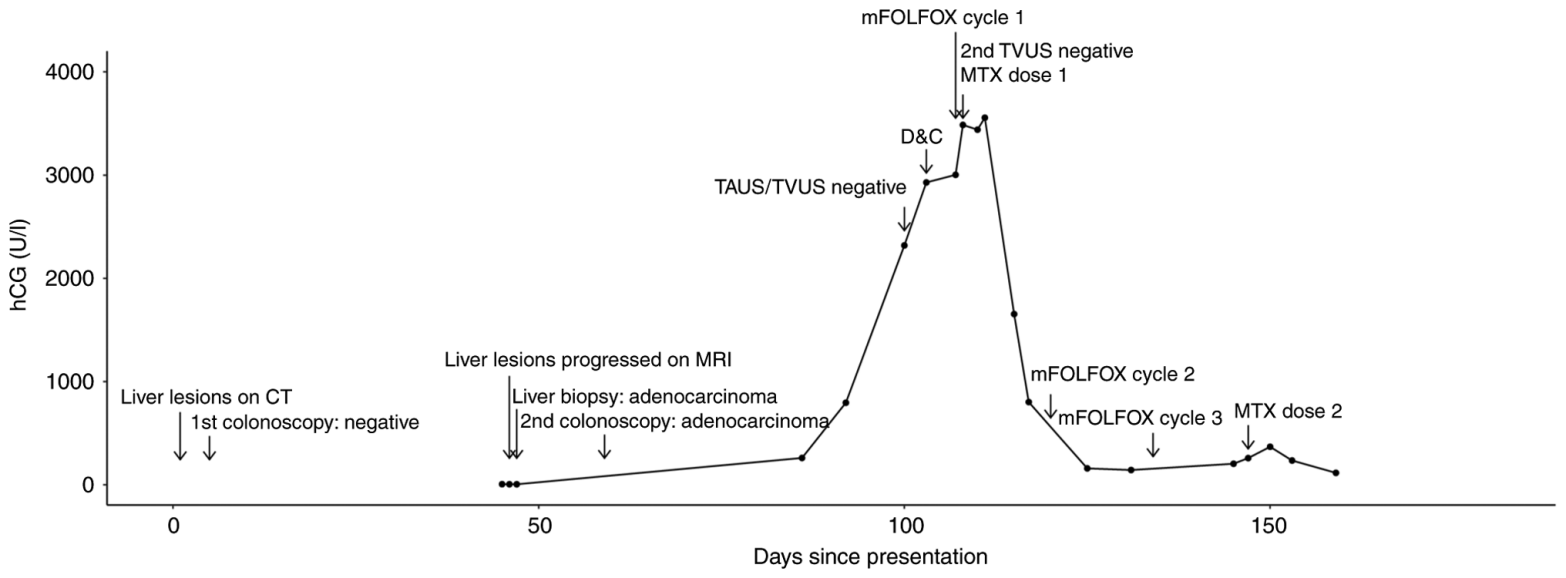
Graph depicting the key events and changes in β-hCG levels in the patient over time. β-hCG, free β subunit of human chorionic gonadotropin; mFOLFOX6, 5-fluorouracil, leucovorin, and oxaliplatin; MTX, methotrexate; TAUS, transabdominal ultrasound; TVUS, transvaginal ultrasound; D&C, dilation and curettage.

The patient was scheduled for chemotherapy with the 5-fluorouracil, leucovorin, and oxaliplatin (mFOLFOX6) regimen, at ~1 month later. As part of the pre-therapy assessment, the analysis of β-hCG was repeated and the results were positive at 260.1 U/l. Transabdominal and transvaginal ultrasound examinations were performed within 2 weeks and neither found intrauterine or ectopic pregnancy. A repeat transvaginal ultrasound following suction dilation and curettage also revealed no intrauterine or ectopic pregnancy. The first dose of mFOLFOX6 was administered 4 days later and methotrexate at 50 mg/m^2^ was also administered intramuscularly the following day. However, the serum β-hCG levels continued to increase, reaching a peak level of 3,556 U/l, 3 days after receiving methotrexate. Previous liver and sigmoid colon biopsy samples were stained for β-hCG. The liver biopsy sample was negative for β-hCG staining, but focal positivity for β-hCG was found in the sigmoid colon sample (data not shown; however, the sample was processed under the same condition). At ~1 month later, the serum β-hCG level of the patient had decreased to 204.1 U/l. The trend of serum β-hCG levels and key events of the patient are summarized in Fig. 2.

## Discussion

Elevated serum β-hCG levels have been reported in 0-20% of patients with colorectal carcinoma, usually at low levels (8). In the case described herein, a woman of reproductive age presented with sigmoid colon adenocarcinoma and was found to have elevated serum β-hCG levels in a range which was concerning for pregnancy. However, no evidence of intrauterine or ectopic pregnancy was found on imaging and curettage, although immunohistochemistry of the colonic adenocarcinoma identified positive β-hCG staining. This finding raised the authors' suspicion of tumor-associated β-hCG production over pregnancy. The patient received mFOLFOX6, as well as methotrexate at around the same time. Soon after, serum β-hCG level markedly decreased.

In a previous study involving 10 cases of β-hCG-positive colorectal adenocarcinoma and 35 β-hCG-negative counterparts, it was reported that β-hCG-positive adenocarcinomas were more likely to occur at the rectosigmoid region and that β-hCG-positive cells were more likely to be distributed at the periphery of the tumor or arranged in clumps resembling syncytiotrophoblasts (9). The expression of β-hCG and themorphology of these cells suggested the possibility of highly invasive, syncytiotrophoblast-like behavior of the adenocarcinoma, which could be a manifestation of the de-differentiation of malignant tissue and could facilitate progression and metastasis.

Of note, 2 cases with unusually high serum β-hCG levels (50,000 and 154,000 mIU/ml) in colon cancer were previously reported in adenocarcinoma of the colon with syncytiotrophoblast-like cells (10,11). Rapid progression and metastasis were reported in both cases. However, no syncytiotrophoblast-like cells were found in the patient in the present study, although the possibility cannot be excluded, since there was no primary resection and endoscopic biopsy may have missed the region with syncytiotrophoblast-like morphology. Serum β-hCG levels in the thousands were also observed in other case reports of colon cancer. Tumor-associated β-hCG production may be associated with sensitivity to chemotherapy and the serum β-hCG level also decreased following the response to chemotherapy (12), which is similar to the response in trophoblastic tumors. Serum β-hCG levels are associated with immunohistochemical β-hCG staining in the tumor samples; however, the correlation is relatively weak, indicating that tumor secretion may not be the only mechanism for elevated serum β-hCG levels (3). A previous study also found that β-hCG-derived peptides can stimulate CD4^+^ and CD8^+^ T-lymphocytes *in vitro* (13), which could partially explain its sensitivity to chemotherapy. However, further studies are required to better understand the underlying mechanisms of such behavior in β-hCG-positive colon cancer with modern techniques, such as single-cell sequencing that analyzes the interaction between cancer cells and their microenvironments (14). Therefore, in patients with colorectal cancer and high serum β-hCG levels, the monitoring of serum β-hCG levels can provide additional information on prognosis and response to therapy. However, larger-scale studies are also required to determine whether routine β-hCG staining on biopsy samples with colorectal adenocarcinoma or routine serum β-hCG testing in newly diagnosed colorectal adenocarcinomas provide additional benefits to patient care. In female patients of reproductive age with malignancy, the analysis of β-hCG, on tumor histology as well as imaging studies for pregnancy need to be obtained to help with differential diagnosis.

In terms of the clinical management, methotrexate was administered in the patient in the present study for an assumed diagnosis of ‘pregnancy of unknown location’ due to a continuously increasing serum β-hCG level in the absence of evidence for intrauterine pregnancy, consistent with recommendations by the American College of Obstetricians and Gynecologists (ACOG) (15), despite the high probability of β-hCG from neoplastic source. However, colorectal adenocarcinoma remains a less known neoplastic cause of elevated serum levels of β-hCG, and on UpToDate (16), which is a key reference for clinical practitioners, only gestational trophoblastic disease is listed on the differential diagnosis as a neoplastic source of β-hCG. In addition, β-hCG is not one of the routine immunohistochemical markers of colorectal cancer pathology. Thus, while waiting for the results of β-hCG staining as an extra pathological exam, methotrexate was administered to prevent a potentially life-threatening condition. Future studies are also required to clarify the benefits and risks of empirical treatment with methotrexate.

In conclusion, in women of reproductive age with colorectal carcinoma, β-hCG cannot only serve as a maker for pregnancy but also as a tumor marker. Positive β-hCG in serum or histology results in patients with colorectal adenocarcinoma are associated with a worse prognosis, increased tissue invasion and metastasis, as well as worse survival outcomes. However, serum β-hCG levels may be associated with the response to treatment. Routine serological and histological β-hCG testing as a tumor marker needs to be considered in patients with suspected or confirmed colon cancer.

## Data Availability

The datasets used and/or analyzed during the current study are available from the corresponding author on reasonable request.
